# The Complex Regulatory Role of Cytomegalovirus Nuclear Egress Protein pUL50 in the Production of Infectious Virus

**DOI:** 10.3390/cells10113119

**Published:** 2021-11-11

**Authors:** Sigrun Häge, Nicole Büscher, Victoria Pakulska, Friedrich Hahn, Annie Adrait, Steffi Krauter, Eva Maria Borst, Ursula Schlötzer-Schrehardt, Yohann Couté, Bodo Plachter, Manfred Marschall

**Affiliations:** 1 Institute for Clinical and Molecular Virology, Friedrich-Alexander University of Erlangen-Nürnberg (FAU), 91054 Erlangen, Germany; friedrich.hahn@uk-erlangen.de; 2 Institute for Virology and Forschungszentrum für Immuntherapie, University Medical Center of the Johannes Gutenberg-University Mainz, 55131 Mainz, Germany; bueschni@uni-mainz.de (N.B.); krauter@uni-mainz.de (S.K.); plachter@uni-mainz.de (B.P.); 3 Institut National de la Santé et de la Recherche Médicale (INSERM), University Grenoble Alpes, CEA, UMR BioSanté U1292, CNRS, CEA, FR2048, 38000 Grenoble, France; victoria.pakulska@cea.fr (V.P.); annie.adrait@cea.fr (A.A.); yohann.coute@cea.fr (Y.C.); 4 Institute of Virology, Hannover Medical School (MHH), 30625 Hannover, Germany; borst.eva@mh-hannover.de; 5 Department of Ophthalmology, University Medical Center Erlangen, FAU, 91054 Erlangen, Germany; Ursula.Schloetzer-Schrehardt@uk-erlangen.de

**Keywords:** human cytomegalovirus, regulation of viral replication, nuclear egress complex (NEC), NEC protein pUL50, functional properties, conditional expression, efficiency of infectious virus production

## Abstract

The regulation of the nucleocytoplasmic release of herpesviral capsids is defined by the process of nuclear egress. Due to their large size, nuclear capsids are unable to traverse via nuclear pores, so that herpesviruses evolved to develop a vesicular transport pathway mediating their transition through both leaflets of the nuclear membrane. This process involves regulatory proteins, which support the local distortion of the nuclear envelope. For human cytomegalovirus (HCMV), the nuclear egress complex (NEC) is determined by the pUL50-pUL53 core that initiates multicomponent assembly with NEC-associated proteins and capsids. Hereby, pUL50 serves as a multi-interacting determinant that recruits several viral and cellular factors by direct and indirect contacts. Recently, we generated an ORF-UL50-deleted recombinant HCMV in pUL50-complementing cells and obtained first indications of putative additional functions of pUL50. In this study, we produced purified ΔUL50 particles under both complementing (ΔUL50C) and non-complementing (ΔUL50N) conditions and performed a phenotypical characterization. Findings were as follows: (i) ΔUL50N particle preparations exhibited a clear replicative defect in qPCR-based infection kinetics compared to ΔUL50C particles; (ii) immuno-EM analysis of ΔUL50C did not reveal major changes in nuclear distribution of pUL53 and lamin A/C; (iii) mass spectrometry-based quantitative proteomics showed a large concordance of protein contents in the NIEP fractions of ΔUL50C and ΔUL50N particles, but virion fraction was close to the detection limit for ΔUL50N; (iv) confocal imaging of viral marker proteins of immediate early (IE) and later phases of ΔUL50N infection indicated a very low number of cells showing an onset of viral lytic protein expression; and, finally (v) quantitative measurements of encapsidated genomes provided evidence for a substantial reduction in the DNA contents in ΔUL50N compared to ΔUL50C particles. In summary, the results point to a complex and important regulatory role of the HCMV nuclear egress protein pUL50 in the maturation of infectious virus.

## 1. Introduction

The human cytomegalovirus (HCMV) is a worldwide distributed β-herpesvirus that is able to persist life-long in its human host. While most of HCMV infections are asymptomatic, immunosuppressed individuals and immunonaïve unborn babies or infants may suffer from severe or even life-threatening sequelae. The HCMV-induced pathogenesis is widely determined by the magnitude of viral reproduction [1]. During lytic replication, the amplification and packaging of the viral genomic DNA into capsids occurs in the nucleus, prior to the final steps of maturation of infectious particles proceeding in the cytoplasm. The transition of capsids from the nucleus into the cytoplasm is the specifically regulated and crucial process of nuclear egress, which is conserved in all herpesviruses. In the case of HCMV, nuclear egress is primarily regulated by the two heterodimerizing viral proteins (pUL50 and pUL53) which colocalize at the nuclear envelope, thereby defining the core nuclear egress complex (NEC). This core NEC recruits several viral and cellular proteins, resulting in a multicomponent NEC, which then promotes the reorganization of the nuclear lamina and provides a docking platform for nuclear capsids. Thus, the regulated nuclear egress mediates the envelopment and transient de-envelopment of genome-packaged C-capsids at the inner or outer nuclear membranes, respectively, during this multistep nucleocytoplasmic transfer of viral capsids. In many aspects, this process is reminiscent of the cellular pathways of vesicle-mediated transport [2]. During intracellular trafficking, the budding and scission of membrane vesicles is meditated by specialized proteins, such as clathrin, which induce cytosolic membrane curvature in order to extrude cytoplasmic vesicles. In addition, vesicle formation can be based on the invagination of the endosomal membrane, as reported for the formation of multivesicular bodies, e.g., exosomes mediating cellular waste disposal [3,4]. Interestingly, a number of studies illustrated that the regulatory principle of herpesviral nuclear capsid egress is not, as initially thought, unique to herpesviruses, but obviously also represents a specific mode of the cellular vesicle-mediated transport that is able to mediate the delivery of various types of cargo through the nuclear envelope [2,5,6,7].

In the case of HCMV nuclear egress, a central mechanistic determinant appears to be given by the multi-interacting protein, pUL50, anchored in the nuclear membrane through its trans-membrane domain. As a key point of functionality, pUL50 is able to recruit its nucleoplasmic counterpart pUL53 to the nuclear rim by a hook-into-groove interaction. Assemblies are formed together with further NEC-associated proteins, in particular the viral kinase pUL97 [8]; cellular kinases, such as CDK1 [9]; the prolyl cis/trans isomerase Pin1 [10]; and a number of additional regulators [11,12]. Specifically, the kinases play a crucial role during nuclear egress by phosphorylation of the lamins, a modification leading to a local reorganization of the nuclear lamina (lamina-depleted areas, LDAs), so that capsids attain access to the inner nuclear membrane. Furthermore, it was shown that pUL50 is phosphorylated by these NEC-associated protein kinases in a site-specific manner. In a recent study, however, we were able to demonstrate that a mutagenesis-based block of these phosphorylation events did not impair viral replication [13].

So far, crystal structures of five core NECs belonging to all three herpesviral subfamilies, α, β, and γ, have been resolved [4,14,15,16,17,18]. Despite a low amino acid sequence identity between the NECs of HSV-1, PrV, VZV, HCMV, and EBV, the structure and the principle of the hook-into-groove interaction was found to be highly conserved [19]. Moreover, the overall functionality of the core NECs is specifically conserved among herpesviruses and, thus, the core NEC proteins might serve as a particularly suitable target for antiherpesviral drugs, either in a selective or a broad-spectrum inhibitory manner. While the essen-tiality of the HCMV core NEC proteins for viral replication has not really been evaluated so far, this issue was specifically addressed for the two α-herpesviruses (HSV-1 and PrV). Data strongly suggested that pUL31 of HSV-1 and pUL31 of PrV (the homologs of HCMV pUL53) are not absolutely essential for nuclear egress [20,21,22]. Indeed, the deletion of ORF-UL31 from the HSV-1 or PrV genomes surprisingly did not lead to a complete block of viral replication, but instead to a relative decrease in viral loads and a partial deficiency in the production of enveloped particles, which could be restored by the use of pUL31-complementing cells. A very similar finding was obtained for the deletion of ORF-UL34 (the homolog of HCMV ORF-UL50) [23,24,25]. Interestingly, an extensive passaging of the pUL31 or pUL34 PrV deletion mutants led to a restoration of loss-of-function variants, and so-called pass mutants, which acquired the ability to produce progeny viruses at wild-type (WT) titers [26,27]. Additional mutations in various viral proteins were found and a virus-induced disintegration of the nuclear envelope, i.e., a pronounced nuclear envelope break down (NEBD), was observed. Furthermore, it was also shown that the NEBD process led to the release of immature and mature capsids into the cytoplasm, suggesting that the core NEC may also function as a quality control checkpoint of nuclear egress [26]. Moreover, experimental indications were provided that mitosis-related processes are involved in herpesvirus-induced NEBD [27]. In the case of the Epstein–Barr virus (EBV), mutants carrying deletions of the coding sequences for nuclear egress proteins BFRF1 and BFLF2 have also been described [28,29]. In both cases, low-level replication of the recombinant viruses was analyzed by the use of complementing cells and those deletion mutants showed strong defects in the efficiency of nuclear egress and primary envelopment. Specifically for the BFRF1-KO virus, the reduction in viral titers was due to sequestration of EBV nucleocapsids in the nuclei of lytically induced cells [28]. In comparison, for the ΔBFLF2 virus, not only a defect in the nucleocytoplasmic egress was observed, but also a defective DNA packaging [29]. As a more specific approach to gain insight into cytomegaloviral NEC proteins, two studies investigated the deletion of either ORF-M50 or ORF-M53, the core homologs of MCMV [30,31]. The genomes of both viral deletion mutants could not be successfully reconstituted to infectious virus stocks, but the defect was rescued by the use of NEC-complementing cells, thus indicating that both core NEC proteins are essential for MCMV replication. In a previous study, we were able to demonstrate that a deletion of ORF-UL50 from the HCMV genome does not block HCMV replication completely, but significantly reduces viral titers [13] in a similar way, as shown for the ΔUL34 mutants of HSV-1 and PrV. Interestingly, in the absence of pUL50 expression, we detected a strong decrease in the production of mature C-type capsids and an accumulation of immature A-type capsids. In our previous reports, the ΔUL50 particles analyzed had commonly been grown on pUL50-complementing cells. However, on the basis that pUL50 is also packaged into virions, at least at very low amounts [32], the previously used ΔUL50 particles may not have completely lacked pUL50.

In the present study, we generated two different versions of ΔUL50 particles, namely those produced in pUL50-complementing cells (ΔUL50C) and those produced in non-complementing cells (ΔUL50N). This comparison was made to gain deeper insight into the functional details and the putative range of regulatory roles exerted by pUL50 during the entire HCMV replication cycle. To this end, the two different virus particle versions were analyzed with regard to (i) their DNA and protein contents; (ii) their infectivity by measuring viral replication kinetics and by phenotypical analysis using pUL50-complementing cells; (iii) the intracellular localization of proteins by immunogold EM studies; (iv) the proteomic composition by Western blot and mass spectrometry-based analyses; (v) the induction of viral immediate early- and later-phase lytic gene expression; and (vi) a substantial reduction in encapsidated genomes in ΔUL50N progeny virus. As a clear result, pronounced differences between the ΔUL50C and ΔUL50N particles were observed with these experimental approaches. Most importantly, data of the study provided points of evidence for a complex and important regulatory role of the HCMV nuclear egress protein pUL50 in the maturation of infectious virions.

## 2. Materials and Methods

### 2.1. Cell Culture and Virus Infection

Primary human foreskin fibroblasts (HFFs, own cell cultures repository of M.M. laboratory) and HFF-UL50 cells [33] were maintained at 37 °C, 5% CO_2_, and 80% humidity in MEM supplemented with 10% fetal bovine serum (FBS, FBS-12A, Capricorn Scientific, Ebsdorfergrund, Germany), 1×GlutaMAX™ (35050038, Thermo Fisher Scientific, Waltham, MA, USA), and 10 µg/ml of gentamicin (22185.03, SERVA, Heidelberg, Germany). For the cultivation of HFF-UL50, tetracycline negative FBS (FBS-TET-12A, Capricorn Scientific) was used and, additionally, 500 µg/ml of geneticin was added (G418, 10131035, Thermo Fisher Scientific). The expression of pUL50 in the HFF-UL50 cells was induced by addition of 500 ng/ml of doxycycline (D9891, Sigma-Aldrich, St. Louis, MO, USA) which was refreshed at least every 3rd day (d). For HCMV infection of HFF-UL50, the cells were induced with dox one d prior to infection to induce pUL50 expression. HFF or HFF-UL50 cells were inoculated with stocks of HCMV AD169 (WT) or AD169-derived recombinant ΔUL50 viruses [33] with equal genome amounts. After incubation for 90 min at 37 °C, the inoculum virus was replaced by fresh medium. Cells or supernatants were harvested or fixed at indicated time points for further analyses.

### 2.2. Purification of ΔUL50 Particles

For the purification of ΔUL50 particles under doxycycline-induced, pUL50-complementing (+dox, ΔUL50C) or uninduced, non-complementing conditions (-dox, ΔUL50N), HFF-UL50 cells were infected with the HCMV ΔUL50 virus (strain AD169) or remained mock-infected. The infection was performed in triplicates for each setting in several T175 flasks. At 4 days post-infection (d p.i.), supernatants were harvested and stored at room temperature and a new medium was added to the cells. At 8 d p.i., supernatants were again harvested and pooled with those from d 4 and centrifuged at 2460 rcf for 20 min to remove the residual cells. For the following ultracentrifugation, 15% sucrose cushions dissolved in VSP buffer (50 mM of Tris–HCL, 12 mM KCl, and 5 mM of EDTA adjusted to pH 7.8) were overlaid with the viral supernatants and centrifuged at 65,000 rcf for 70 min. The virion pellets from each flask corresponding to one triplicate were then resuspended in a VSP buffer without EDTA, and then pooled and separated to aliquots. A few aliquots were taken aside to determine the protein concentration by a BCA assay, whereas all other aliquots were adjusted with EDTA to the EDTA concentration of the VSP buffer and stored at −80 °C before use in further analyses. The protein concentration was determined using the Pierce™ BCA Protein Assay Kit (Thermo Fisher Scientific). For ReadyBlue™ (Sigma-Aldrich) protein staining and Western blot analysis, purified virions were mixed with Laemmli buffer and heat-denaturated at 95 °C for 10 min. An additional purification of ΔUL50C and ΔUL50N particles, specifically for mass spectrometry (MS)-based proteomic analysis, was performed, as described previously [32]. HFF-UL50 cells were treated with or without dox and infected as described above. The protein composition of purified virions was analyzed by SDS-PAGE and silver staining, according to the manufacturer’s protocol.

### 2.3. Quantitative Real-Time PCR (qPCR)

The viral genome copy number in purified virus aliquots or cell culture supernatants was determined by IE1-specific quantitative real-time PCR (qPCR), as described previously [34]. For viral replication kinetics, cells were infected with equal genome amounts and supernatants were collected at the indicated time points. DNase treatment of virus aliquots was performed according to the manufacturer’s protocol (04716728001, Sigma Aldrich). The PCR standard, containing 10^2^ HCMV DNA copies, reached the cycle threshold at cycle number 38 and was on this basis defined as the limit of detection.

### 2.4. Immunogold Labeling and Transmission Electron Microscopy (Immuno-EM)

For immunogold staining of cellular and viral proteins, HFF-UL50 cells were seeded in T75 flasks, which were then either induced with dox or left uninduced, and infected with purified ΔUL50C particles. At 6 d p.i., cells were carefully trypsinized and centrifuged at 106 rcf for 3 min. Pellets were washed with PBS and centrifuged at 68 rcf for 5 min prior to fixation in 4% paraformaldehyde and 0.1% glutaraldehyde in 0.1 M cacodylate buffer (pH 7.4) for 5 h at 4 °C. Specimens were dehydrated serially to 70% ethanol at −20 °C and embedded in resin (LR White; Electron Microscopy Sciences). Ultrathin sections were successively incubated in Tris-buffered saline (TBS), as well as 0.05 M of glycine, 0.5% ovalbumin, and 0.5% fish gelatin. Primary antibodies were diluted in TBS–ovalbumin overnight at 4 °C, and, finally, 10-nm gold-conjugated secondary antibodies (Bio-Cell) were diluted 1:30 in TBS–ovalbumin for 1 h. After rinsing with TBS, the sections were stained with uranyl acetate and examined with a transmission electron microscope (906E; Zeiss Microscopy). Antibodies used for immunogold staining were mAb-lamin A/C (ab108595, Abcam, Cambridge, UK), pAb-lamin A/C pSer22 (ABIN 1532183, Antibodies online, Aachen, Germany), and pAb-UL53 (kindly provided by P. Dal Monte, Bologna, Italy).

### 2.5. Western Blot (Wb) Analysis

SDS-PAGE separation and Wb analysis of virus particles was performed using equal protein amounts, as described previously [35]. Antibodies used for staining were mAb-MCP 28-4, mAb-SCP, mAb-pp28, mAb-pp150 (kindly provided by W. Britt, Birmingham, AL, USA), mAb-pp65, pAb-pp71 (kindly provided by T. Stamminger, Ulm, Germany), and mAb-gB (kindly provided by M. Mach, Erlangen, Germany).

### 2.6. Mass Spectrometry (MS)-Based Proteomic Analyses

Proteins denatured in Laemmli buffer were loaded onto a 4–12% NuPAGE gel (Invitrogen). Three biological replicates were analyzed for each sample type and growth condition. After staining with R-250 Coomassie Blue (Bio-Rad), proteins were digested in gel using trypsin (modified, sequencing purity, Promega), as described previously [36]. The resulting peptides were analyzed with online nano-liquid chromatography coupled to MS/MS (Ultimate 3000 RSLCnano and Q-Exactive HF, Thermo Fisher Scientific) using a 140-min gradient. For this purpose, the peptides were sampled on a pre-column (300 μm × 5 mm PepMap C18, Thermo Scientific) and separated in a 75 μm × 250 mm C18 column (Reprosil-Pur 120 C18-AQ, 1.9 μm, Dr. Maisch). The MS and MS/MS data were acquired by Xcalibur (Thermo Fisher Scientific).

Peptides and proteins were identified by Mascot (version 2.7.0.1, Matrix Science) through concomitant searches against the Uniprot database (Homo sapiens and Human cytomegalovirus taxonomies, June 2021 version), and an in-house developed database containing the sequences of classical contaminant proteins were found in proteomicanalyses (human keratins, trypsin, etc.). Trypsin/P was chosen as the enzyme and two missed cleavages were allowed. Precursor and fragment mass error tolerances were set at 10 and 20 ppm, respectively. Peptide modifications allowed during the search were: carbamidomethyl (C, fixed), acetyl (protein N-term, variable), and oxidation (M, variable). The Proline software [37] was used for the compilation, grouping, and filtering of the results (conservation of rank 1 peptides, peptide length ≥6 amino acids, peptide score ≥25, and false discovery rate of peptide-spectrum match identifications <1% [38], and minimum of one specific peptide per identified protein group). Proline was then used to perform a compilation, grouping, and MS1 quantitation of the identified protein groups based on razor and specific peptides.

Statistical analysis was performed using the ProStaR software [39]. Proteins identified in the contaminant database, proteins identified by MS/MS in less than two replicates of one condition, and proteins detected in less than three replicates of one condition were removed. After log2 transformation, abundance values were normalized by the mean of MCP and TRX1 abundances, before missing value imputation (slsa algorithm for partially observed values in the condition and DetQuantile algorithm for totally absent values in the condition). Statistical testing was conducted with limma, whereby differentially expressed proteins were sorted using a log2 (fold change) cut-off of 1 and a *p*-value cut-off of 0.01, allowing to reach a false discovery rate <5%, according to the Benjamini–Hochberg method. Intensity-based absolute quantitation (iBAQ, [40]) values were calculated from raw MS1 intensities of razor and specific peptides. For each sample, iBAQ values were normalized by the mean of MCP and TRX1 iBAQ values before averaging the values of the three replicates to generate the final iBAQ value of each sample type and condition.

### 2.7. Indirect Immunofluorescence (IF) Analysis and Confocal Laser-Scanning Microscopy

HFF-UL50 cells, either induced or uninduced, were grown on coverslips and infected with ΔUL50C or ΔUL50N particles adjusted to equal genome amounts. At indicated time points after virus adsorption, cells were fixed and stained, as described previously [13], and analyzed using a TCS SP5 confocal laser-scanning microscope (Leica Microsystems, Wetzlar, Germany). Images were processed using the LAS AF software (Leica Microsystems) and Photoshop CS5 (Adobe Inc., San José, CA, USA). Primary and secondary antibodies used for staining were mAb-pp65 (kindly provided by T. Stamminger, Ulm, Germany), anti-Cytomegalovirus-Alexa Fluor 488 (MAB810X, Merck, Darmstadt, Germany), mAb-pp28, mAb-pp150 (kindly provided by William Britt, University of Alabama, Birmingham, AL, USA), mAb-UL44 (kindly provided by Bodo Plachter, University of Mainz, Mainz, Germany), and anti-mouse Alexa 555 (A21422, Thermo Fisher Scientific).

## 3. Results and Discussion

### 3.1. The Propagation and Particle Purification of ORF-UL50-Deleted HCMV by the Use of Complementing HFF-UL50 Cells 

**Figure 1:**
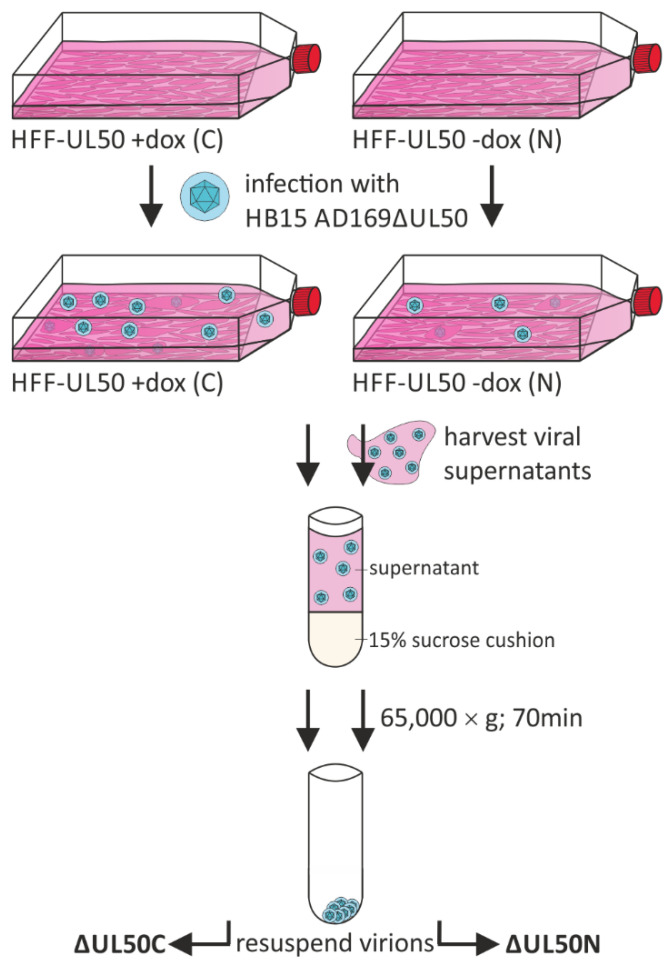
Schematic depiction of the purification procedure of ΔUL50 particles. HFF-UL50 cells, either dox-induced or uninduced were infected with HCMV ΔUL50. Viral supernatants were harvested at 4 and 8 d p.i. and used for particle purification by ultracentrifugation through a sucrose cushion. ΔUL50C particles, derived from pUL50-complementing conditions, and ΔUL50N, from non-complementing conditions, were resuspended in VSP buffer and stored at −80 °C. For each type of conditions, ΔUL50C or ΔUL50N, three comparable replicates of the particle preparations (samples 1, 2, 3; see Figure S1) were generated.

**Figure 2:**
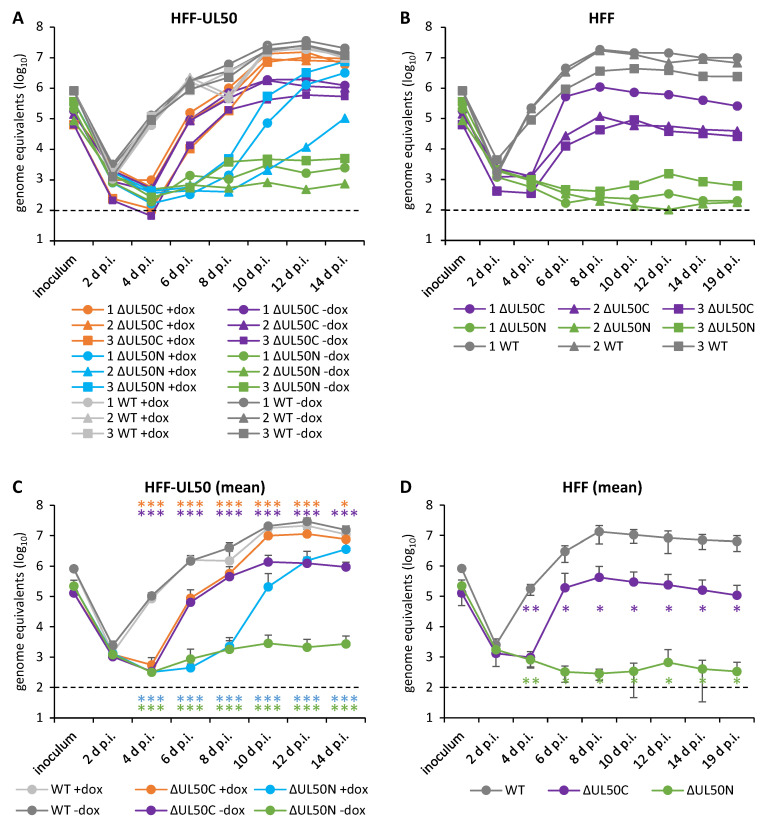
Replication kinetics of ΔUL50C and ΔUL50N show, in both cases, an impairment of replication efficiency in the absence of co-expressed pUL50. Infections were performed with the purified HCMV ΔUL50C compared to ΔUL50N particles, or parental HCMV AD169 (WT), using identical amounts of viral genomes contained in the inocula. HFF-UL50 cells were used, either in the induced (+dox) or uninduced (-dox) state of pUL50 expression, with values presented for the three individual purification replicates, labeled 1, 2, 3 (**A**), or as a mean of 1–3 (**C**). HFFs were used in analogous settings in parallel, also presented as individual values (**B**) or as a mean of 1–3 (**D**). IE1-specific qPCR was applied in duplicate measurements to detect the viral genome equivalents in supernatants harvested at the indicated time points. All infections were performed in triplicates, and mean values + SD are given in the curves. The standard containing 10^2^ HCMV DNA copies reached the cycle threshold at cycle 38 and was, therefore, defined as the limit of detection (black dashed lines). Statistical significance of the values of genome equivalents was calculated by ANOVA (*, *p* ≤ 0.05; **, *p* ≤ 0.01; ***, *p* ≤ 0.001) for 2–19 d p.i. in relation to WT (for panel C, mean value of WT +dox and WT -dox).

**Figure 3:**
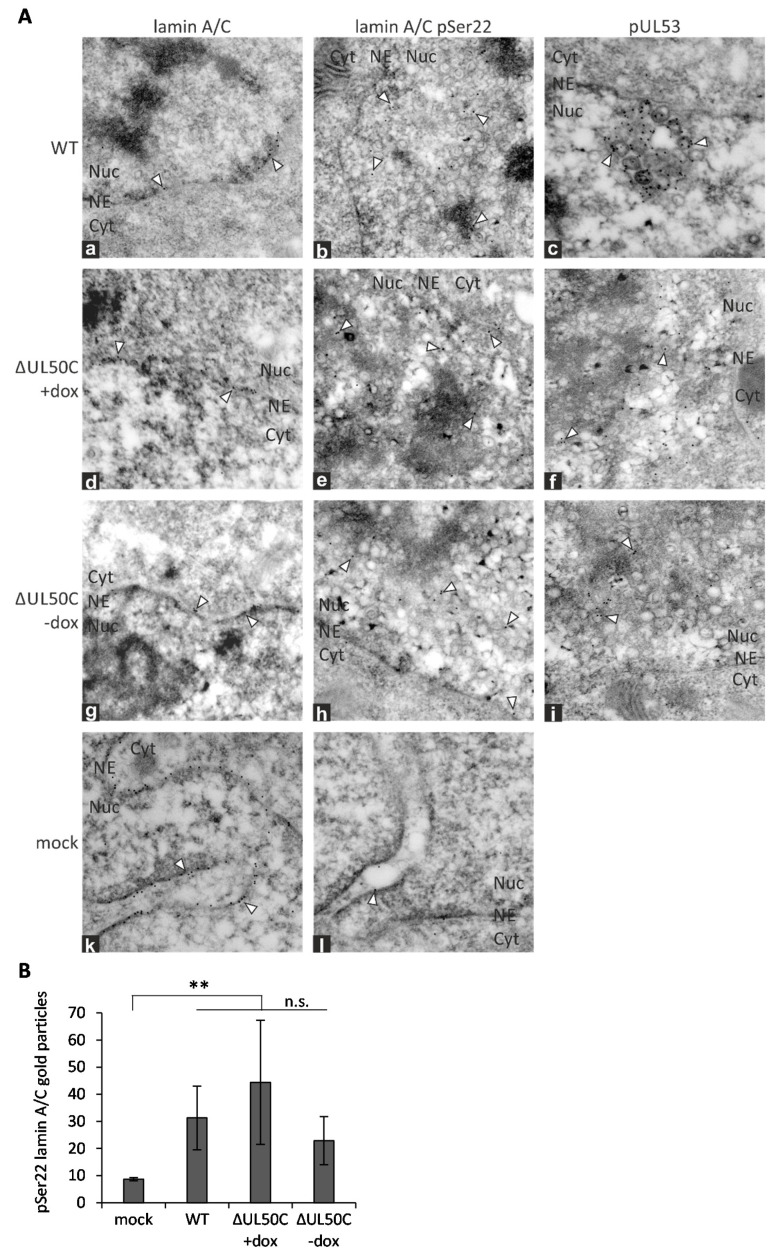
Immunogold EM analysis of WT- and ΔUL50C virus-infected cells. (**A**) HFF-UL50 +dox or −dox infected with WT (**a**–**c**) or ΔUL50C (**d**–**i**) or remained mock-infected (**k**–**l**) (identical amounts of viral genomes contained in the inocula). At 6 d p.i., cells were fixed, and were subjected to sectioning and immunogold staining before analysis by EM. Representative nuclei of cells stained against lamin A/C, pSer22 phosphorylation-specific lamin A/C or pUL53, respectively. Individual gold particles were exemplarily marked by arrowheads. NE, nuclear envelope; Cyt, cytoplasm; Nuc, nucleus. (**B**) Quantitation of the pSer22-specific lamin A/C signals by counting the gold particles in duplicates each in at least two nuclear sections. Mean values ± SD are given, significance was calculated by the Student’s *t*-test (**, *p* ≤ 0.01; n.s., no significant difference between the three indicated settings of infection).

**Figure 4:**
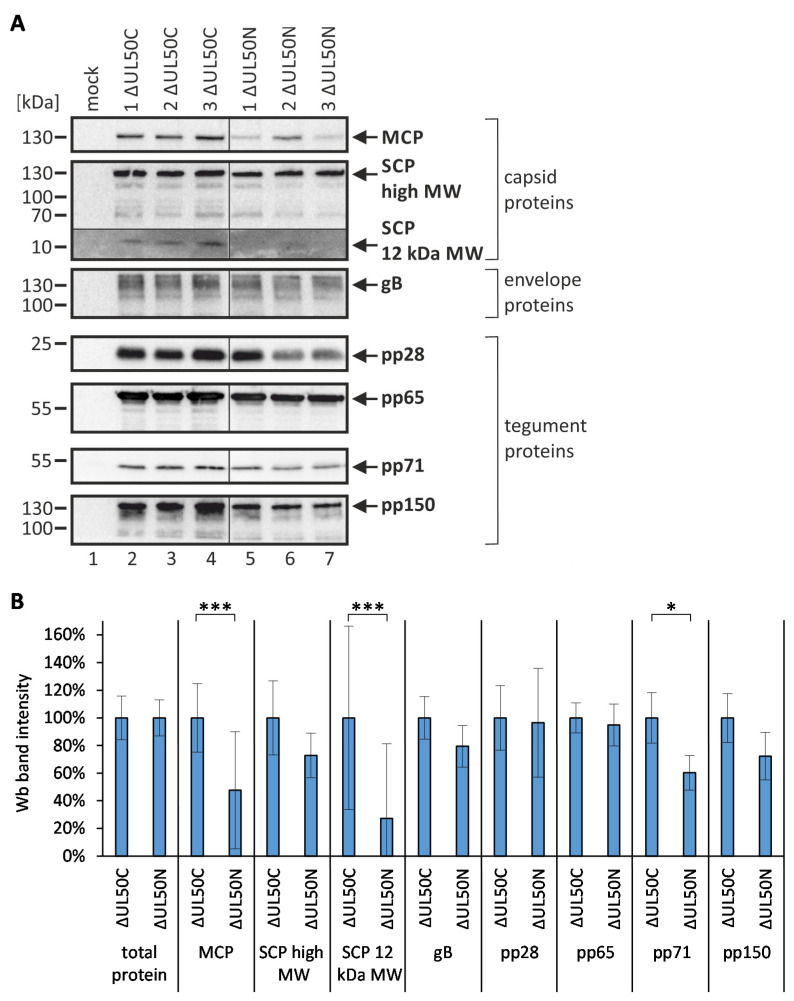
Use of Wb detection as the basis for an initial analysis of the protein composition of ΔUL50C and ΔUL50N particles. (**A**) Wb analysis of ΔUL50C and ΔUL50N particles, adjusted to 5 μg each, immunostained against viral capsid, envelope, and tegument proteins. (**B**) Densitometry-based quantitation, measurements in duplicate, of Wb band intensities using two independent Wb membranes in each case. Total protein was calculated as the sum of densitometry signals for all proteins determined. Mean values ± SD are given, and significance was calculated by the Student’s *t*-test (*, *p* ≤ 0.05; ***, *p* ≤ 0.001).

In order to address the NEC-specific functionality of pUL50, or possibly even wider-ranging activities within the HCMV replication cycle, a recombinant viral genome carrying a deletion of the entire ORF-UL50 was generated by two-step markerless BACmid technology [33]. HCMV AD169ΔUL50 was reconstituted by the use of complementing HFF-UL50 cells, in which the expression of pUL50 is under control of a tetracycline operator and is, thus, specifically inducible with doxycycline (dox) [33]. In our previous reports, all virus stocks used for the experimentation were prepared on complementing HFF-UL50 cells [33]. Here, virus propagation was either performed under the pUL50-complementing conditions (C, +dox), termed ΔUL50C, or at a much lower efficiency under non-complementing conditions (N, -dox), termed ΔUL50N. For large-scale preparations and purification of ΔUL50 particles, viral supernatants were harvested from infected HFFs at 4 and 8 d p.i. (Figure 1). Viral particles were purified by ultracentrifugation using a sucrose cushion, as to be analyzed in the experiments described by Figure 2, Figure 3 and Figure 4 and Figures 6 and 7. Subsequently, the viral ΔUL50C and ΔUL50N particles were resuspended in VSP buffer and aliquots were stored at −80 °C. The purification procedures were performed in triplicates for both types of culture conditions, additionally including culture supernatants of mock-infected cells as a negative control.

For further analyses, the contents of viral proteins and genomes in purified ΔUL50 particles were analyzed (Figure S1). The protein concentration of each replicate of the triplicate purification settings was quantified by the BCA assay. Samples of the particles, equally adjusted according to the measured protein concentrations (Figure S1C), were subjected to SDS-PAGE and Western blot (Wb) analysis (Figure S1A,B). The Instant Blue staining of the SDS-PAGE gel revealed a similar pattern for both the ΔUL50C and ΔUL50N particles (Figure S1A). In the mock controls, a band was additionally detectable, most probably representing the 69-kDa large bovine serum albumin, a major component of the cell culture media, thus indicating some degree of additional presence of cellular proteins. In parallel, the Wb staining verified the presence of viral proteins MCP, i.e., pp65 and pp28 (Figure S1B). It should be mentioned that the particle preparations applied in this study, i.e., sucrose cushions and glycerol–tartrate gradients, contained a huge amount of viral dense bodies (DBs) in the fractions that may have some additional impact on the measurements. Here, the sucrose cushion was used as a facile protocol to pellet the particles. Since these preparations generally represent mixtures of virions, as well as noninfectious NIEPs and DBs, a glycerol–tartrate gradient protocol was additionally used for more detailed aspects of the study (see below). Interestingly, it seemed at this stage of investigation that MCP as well as pp28 were decreased in ΔUL50N particles compared to ΔUL50C. To analyze the levels of viral genomes in the purified particles, qPCR analysis was performed. Of note, there were some slight differences comparing the three replicates of ΔUL50C or ΔUL50N. However, and more importantly, there was a substantial difference between the two particle preparations, as indicated by a 9.6-fold higher viral genome content in ΔUL50C compared to ΔUL50N (Figure S1C). In addition, the mean protein concentration of the ΔUL50C samples was 2.4-fold higher than that of ΔUL50N. In summary, the ΔUL50N particles, derived from non-complementing cells, exhibited a somewhat decreased amount of total protein in the preparations compared to ΔUL50C. In addition, considered as an even more relevant difference, a strongly decreased amount of viral genomes was detected.

### 3.2. Determination of the ΔUL50 Virus Replication Characteristics under Conditions of pUL50 Complementation versus Non-Complementation

To investigate the replication kinetics of the purified ΔUL50C and ΔUL50N particles, HFF-UL50 +dox or -dox, as well as normal HFFs, were used for infection. The viral inocula had been adjusted to identical levels of viral genomes (Figure 2). After the harvest of culture supernatants at the indicated time points, IE1-specific qPCR was applied to determine viral genome copy numbers. The resulting replication kinetics, representing the samples 1, 2, and 3 of the purification replicates, were based on triplicates of infection performed in a 24-well format, and on qPCR measurements additionally performed in duplicates. Used as a reference control, WT infection was not markedly influenced by the induction of pUL50 expression as expected (Figure 2A,C). Infection of HFF-UL50 cells with either of the recombinant virus preparations showed a slight delay compared to WT, whereby the delay for ΔUL50N was much more drastic than for ΔUL50C (Figure 2A,C). Markedly, the replication curves of ΔUL50N under -dox conditions remained almost at background levels along the investigated time course up to 14 d p.i. For ΔUL50C, replication efficiency almost reached WT levels in the pUL50-complementing setting, while the non-complementing setting remained approx. one log lower than WT throughout the investigated range of time. Notably, when comparing ΔUL50C with ΔUL50N (Figure 2C), the difference was dramatic at 6 d and 8 d p.i., but not at 4 d p.i. This interesting aspect may possibly arise from a general delay of ΔUL50 preparations versus WT, on the one hand, but also a comparably very low number of cells lytically infected in the case of ΔUL50N, on the other hand, thus producing an increasing difference between ΔUL50N and ΔUL50C seen at the later time points of 6 d and 8 d p.i. While ΔUL50N then showed a pronounced rise of replication under +dox conditions at time points between 8 d to 14 d p.i., finally attaining the level of ΔUL50C, this was not the case for -dox conditions of ΔUL50N. The theoretical option that pUL50 in the incoming virions of ΔUL50C might additionally regulate the stronger onset of HCMV replication compared to ΔUL50N, as an alternative explanation to the rate-limiting low number of cells initially infected with ΔUL50N, is not considered very probable, since the presence of virion-associated pUL50 has generally been restricted to a very low quantity, remaining around the detection limit [32].

These findings were confirmed in the parallel setting using normal HFFs (Figure 2B,D). Specifically, the replication of ΔUL50N in normal HFFs was completely blocked. This is interesting considering that the ΔUL50N was not found completely inactive under -dox conditions in HFF-UL50 (Figure 2A,C), but the detectable viral genomes remained at a very low, almost background, level, with only some slight transient increases. Referring to the latter point, there is a theoretical option that some residual expression of pUL50 might occur under -dox conditions; however, in practice, this has been mostly ruled out by the use of antibiotics-free FBS for the cultivation of complementing cells (for details of protocol optimization, see [33]). Independent from this, the ΔUL50C preparations showed a clearly different phenotype, in that replication in normal HFFs occurred at an intermediate level, thus indicating an impairment of replication, but not a fully inactive state. This characteristic of the ΔUL50 virus has been reported before [13]. Thus, the present data confirmed that co-expressed pUL50 is able to rescue the phenotype of both ΔUL50C and ΔUL50N particles, at least in a delayed manner. In comparison, the absence of pUL50 led to an approximately one-log reduced level of viral genomes in ΔUL50C infection and an even lower level in ΔUL50N infection. Together, these data suggest that particles derived from both +dox and -dox conditions represent basically functional particles, but are drastically varying in their degree of infectivity. While the complementation with pUL50 compensates these differences, the lack of pUL50 severely impairs infectivity and replication, thus strengthening the statement that pUL50 is an important determinant of HCMV replication efficiency.

### 3.3. Immunogold EM Analysis of Viral Capsids, Egress Regulator pUL53 and Proteins of the Nuclear Lamina in ΔUL50-Infected Fibroblasts

In order to analyze protein localization characteristics of the ΔUL50 virus in a high resolution, immunogold EM analysis was performed (Figure 3). For this purpose, HFF-UL50 cells under +dox or -dox conditions were infected with either WT or ΔUL50C virus. In separate settings, pUL53, lamin A/C, and pSer22 phosphorylation-specific lamin A/C were used for immunogold stainings and subsequent transmission EM analysis. As previously described by our group, a typical thinning of the lamina, mediated by site-specific phosphorylation and a subsequently induced lamin A/C reorganization [10,41,42], could likewise be observed in the HCMV-infected cells compared to the non-infected mock control (Figure 3A, comparing a, d, and g with k). Of note, signals for lamin A/C pSer22 were not exclusively found at the nuclear rim but also in a more diffuse location within the nucleoplasm. Furthermore, there was also a clear intranuclear association of pUL53 with viral capsids, either in the proximity or at a distance from the nuclear rim, as described earlier [41]. Interestingly, no substantial differences were observed between WT and ΔUL50C infections, neither in the +dox induced nor in the -dox uninduced state. A quantitation of signals by microscopic counting indicated a significant increase of Ser22-specific lamin A/C phosphorylation in the HCMV-infected compared to mock-infected cells, but there was no significant difference between WT and ΔUL50C (Figure 3B). Taken together, the ΔUL50C mutant did not show a detectable alteration in patterns of the investigated proteins in comparison to WT.

### 3.4. Initial Qualitative and Semi-Quantitative Assessment of ΔUL50 Particle Preparations by the Use of Wb Analysis

For an initial qualitative and semi-quantitative assessment of protein contents in the ΔUL50 particle preparations described above, identical amounts of 5 μg each were subjected to Wb and immunostaining against the indicated viral proteins (Figure 4A). In particular, the major and the smallest capsid proteins (MCP and SCP), the envelope glycoprotein B (gB), and four tegument proteins (pp28, pp65, pp71, and pp150) were applied to Wb immunostaining. On the qualitative basis, all proteins were detectable in each of the three replicates investigated. Considering a first quantitative estimate, however, some of these proteins appeared weaker in the detection signal when comparing ΔUL50N with ΔUL50C, as especially indicated by MCP, SCP, pp28, and pp71. Notably, the staining of the low molecular weight band of SCP, approx. 12 kDa, was very weak around the detection limit, while the larger SCP variety of approx. 130 kDa, which has been described earlier by the use of our sensitive mAb-SCP [41], was detected at a more constant level. To quantitate these results, two separately prepared Wb membranes from these samples were subjected to densitometry using the Aida Image Analyzer (Figure 4B). This analysis revealed a rather constant level for most of the viral proteins analyzed, but some decrease for others, which was found significant for MCP, SCP-12 kDa, and pp71 in ΔUL50N particles compared to ΔUL50C. However, at this stage, it could not be ruled out that such differences seen on Wb were due to the normalization of viral infection inocula based on total protein amounts. Nevertheless, this initial estimate of protein quantities showed a general basis of protein conformity, but also indicated some examples of possible differences between the two particle versions, which had to be further investigated on a confirmatory level of mass spectrometry.

### 3.5. Detailed Qualitative and Quantitative Assessment of ΔUL50 Particle Preparations by the Use of MS-Based Proteomics

**Figure 5:**
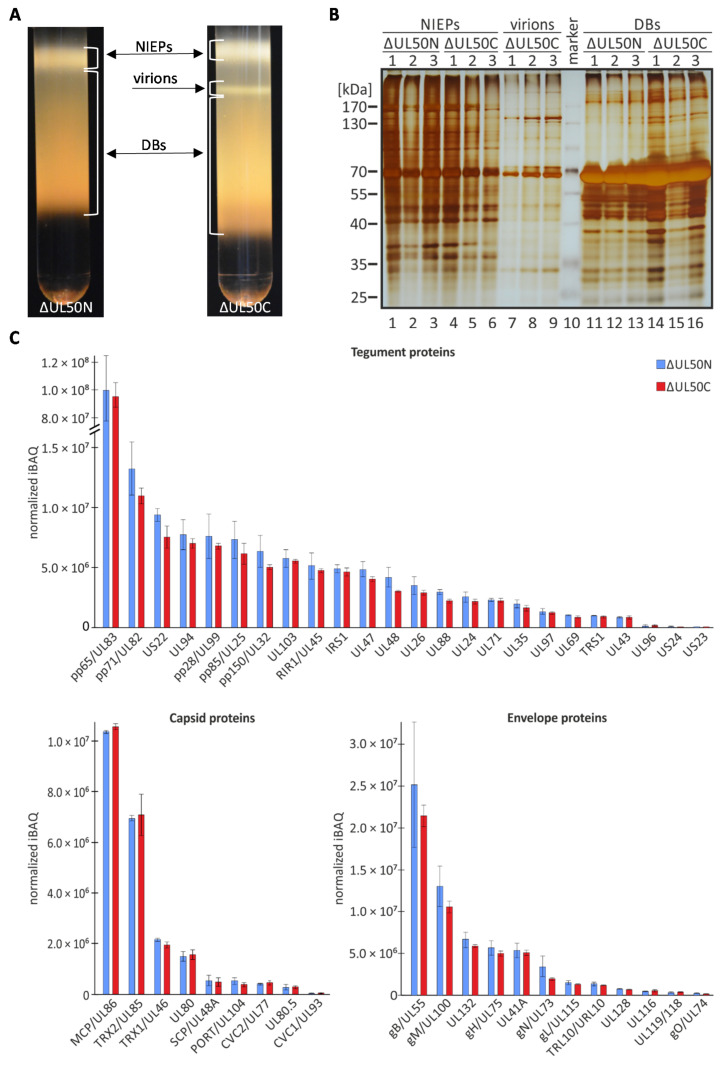
MS-based quantitative analysis of gradient-purified ΔUL50C and ΔUL50N NIEPS. (**A**) Purification of the ΔUL50 virus grown on pUL50-complementing (ΔUL50C) or non-complementing cells (ΔUL50N) by glycerol–tartrate gradients resulting in three different fractions, i.e., dense bodies (DBs), noninfectious enveloped particles (NIEPs), and virions. Note that the absence of a detectable virion band, however, did not rule out a minor fraction of infectious virions in the preparations (see Figure 2). (**B**) Silver staining of the purified DB, NIEP, and virion fractions. (**C**) MS-based quantitative analysis of ΔUL50N and ΔUL50C NIEP fractions. The abundance of the detected proteins was calculated as the averaged and extracted intensity-based absolute quantitation (iBAQ) value, normalized to the mean of MCP and TRX1 iBAQ values. Mean values of biological triplicates ± SD are given and proteins were categorized into the three localization subclasses tegument, capsid, and envelope.

Next, a more sophisticated investigation of viral proteins contained in particles was performed by applying a highly sensitive approach of MS-based proteomics. For this purpose, larger volumes of 140–300 ml of infected-cell supernatants were utilized for a procedure of glycerol–tartrate gradient ultracentrifugation [32,43,44]. Using this procedure that proved to be highly reliable in previous studies [32,43], the three particle fractions released from HCMV-infected HFF-UL50 +dox or -dox were separated. These were virions, noninfectious enveloped particles (NIEPs, representing enveloped, genome-free B capsids differing from virions by the presence of scaffolding proteins UL80 and UL80.5), and dense bodies (DBs, representing spheroidal aggregates mainly composed of the tegument protein pp65, but lacking capsids) [45,46]. The particle fractions were illuminated by light scattering (Figure 5A). It became evident that the virion fraction was exclusively detectable for the ΔUL50C preparations, but was absent or below the detection limit for ΔUL50N. This finding was surprising based on the fact that our previous experimentation, as performed with the initial sucrose cushion-purified particles, already showed a detectable viral infectivity in the form of replication kinetics (see Figure 2). This indicated a low quantity of infectious virions in ΔUL50N preparations, which was not detectable in our gradient purification procedure. In both preparations, however, large amounts of the two noninfectious particle types were found, namely NIEPs and DBs. A closer inspection of these preparations by SDS-PAGE/silver staining (Figure 5B) revealed a mostly normal content of viral proteins. The NIEP fractions showed a high background of additional protein bands, which were attributable to cell debris and serum protein oligomers originating from the medium, banding on top of the gradient together with NIEPs. As expected, the DBs showed the predominant 70-kDa band, containing the abundant pp65 tegument protein. The virion and NIEP fractions were then subjected to MS-based label-free quantitative proteomic characterization (Tables S1 and S2). For the virion fractions of ΔUL50C, a viral protein composition was found comparable to data of WT particles reported earlier (Table S2, [32]). Since no detectable virion fraction could be purified from ΔUL50N, we focused on the comparison of NIEPs protein content. These particles differ from the composition of virions solely by the presence of the assembly protein UL80/UL80.5 and the lack of viral DNA. Concerning the NIEP fractions of ΔUL50C and ΔUL50N preparations, extracted quantitative values of each protein in the different samples were normalized towards the basis of both major capsid protein (MCP) and the triplex capsid protein 1 (TRX1). The average abundance of these proteins was considered stable between the different samples analyzed (n = 3 for ΔUL50C and ΔUL50N). A selection of viral tegument, capsid, and envelope proteins is presented (Figure 5C) for ΔUL50C (red bars) and ΔUL50N (blue bars). Interestingly, none of the 84 detected viral proteins was reduced or increased in the ΔUL50N NIEPs compared to ΔUL50C (Table 1 and Table S1). Thus, the absence of pUL50 during the generation of ΔUL50N particles obviously did not affect the overall protein assembly in the form of NIEPs, but reduced the production of infectious virions to levels undetectable in the gradient (see Figure 5A). It appears striking that no significant difference for any of the scored proteins was detectable between ΔUL50N and ΔUL50C. The lack of virions in the ΔUL50N preparations was highly compatible with our earlier finding that the ΔUL50 virus shows a very pronounced deficiency in the nuclear formation of genome-packaged C-type capsids [13]. Thus, the analysis of ΔUL50 particles showed that, when produced under non-complementing conditions, the production of ΔUL50 virions was completely lacking or drastically impaired, whereas the assembly of the NIEPs concerning protein composition was not affected.

### 3.6. Confocal Imaging-Based Measurement of the Kinetics of ΔUL50 Viral Onset of Immediate Early Gene Expression 

**Figure 6:**
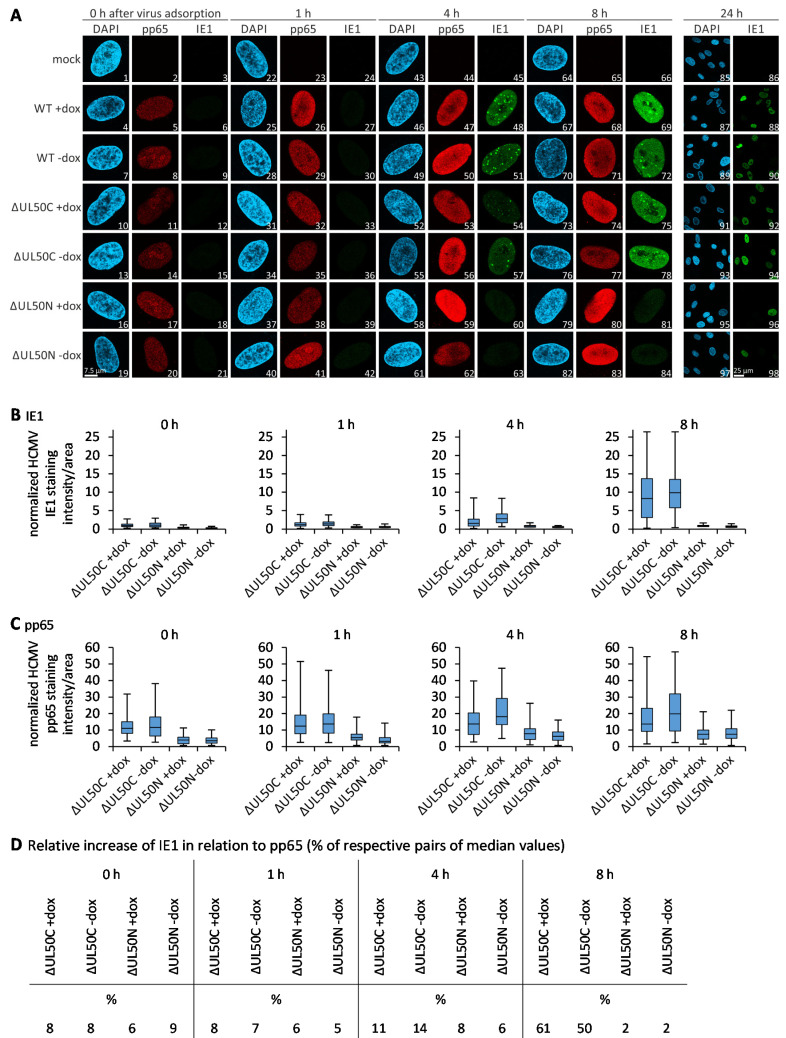
Activation of IE1 expression in ΔUL50C- and ΔUL50N-infected HFF-UL50. (**A**) HFF-UL50, either induced or uninduced, were infected with WT, ΔUL50C, or ΔUL50N, adjusted to identical viral genomic loads. After 1.5 h of virus adsorption, cells were fixed at the indicated time points and used for immunostaining with virus-specific antibodies and subsequent analysis by confocal imaging. The nuclei were counterstained by DAPI, and a scale bar is given in 19. Quantitation of the signal intensities of IE1 (**B**) and pp65 (**C**) as normalized to the total protein concentrations of virus inocula used for infection. The evaluation of 78 cells for each setting is depicted in box plots. Centre lines represent the medians, box limits indicate the 25th and 75th percentiles, and whiskers range from the minimum to the maximum of all data. (**D**) Median values of IE1 signal intensities in relation to the respective pp65 signals in %.

The data collected so far hinted to the point that the absence of pUL50 during the production of ΔUL50N may lead to viral particles that are devoid in full functionality and infectivity. To further investigate this aspect, we focused on the activation of viral IE1 gene expression as an indicator of the onset of lytic replication [47]. HFF-UL50 cells were cultivated, either under +dox induced or -dox uninduced conditions, and were then infected with WT, ΔUL50C, or ΔUL50N virus, as adjusted to identical viral genomic loads of the inocula. At the indicated immediate early time points p.i., cells were harvested, and the expression of viral IE1 protein was analyzed by IF staining and subsequent confocal imaging (Figure 6A). The activation of IE1 expression, already initiated by the entry-conferred import of particle-associated tegument protein pp71 [48,49], is one of the first steps of lytic viral replication. In this experiment, the import of the most abundant tegument protein pp65 was taken as a constant marker of viral entry, and the onset of IE1 expression was analyzed as a function of time after virus adsorption (Figure 6A). While the pp65 signal became detectable in all settings, directly with the addition of viral inocula, i.e., 0 h after virus adsorption, a steady increase in IE1 was noted with the time of investigation, i.e., 1–8 h after adsorption. Importantly, in WT- and ΔUL50C virus-infected cells, IE1 expression already started at 4 h p.i. Whereas, at the time points of 4–8 h p.i., no activation of IE1 was found for ΔUL50N. Of specific interest was the finding that even under conditions of +dox induction, the pUL50-complementing cells could not compensate this IE1 deficiency of the ΔUL50N virus. Surprisingly, a more detailed investigation revealed that, in the ΔUL50N-infected cells, IE1-expressing cells were also detectable; however, this was a very rare event compared to the WT- and ΔUL50C-infected cells (Figure 6, panel 24 h). The findings suggest that viral particles produced in the absence of pUL50 are mostly incapable of inducing IE protein expression. 

To illustrate this deficiency of viral IE1 immediate onset, the signal levels were quantified on a single-cell basis by microscopic counting using the AIDA Image Analyzer (Figure 6B,C; in addition, the distribution and variance of the individual values of the measurements are provided by histograms in Figure S2). On this basis, IE1 and pp65 signal quantitations were normalized according to the total protein concentrations of virus inocula used for infection. The resulting intensities were depicted in a box plot conveying the variation of signals within cell populations. This quantitation indicated that the signal intensities for IE1 expression were comparable between +dox induced and -dox uninduced cells infected with ΔUL50C, whereas it was nearly undetectable for ΔUL50N under both conditions (Figure 6B). Furthermore, it could be shown that more pp65 was also present in ΔUL50C-infected cells compared to ΔUL50N (Figure 6C). Finally, the median values obtained for IE1 were pairwise set in relation to pp65, i.e., each value of IE1 in percentage to the respective value of pp65, to display the relative increase in IE1 (Figure 6D). This measurement revealed at the very early time points of 0–1 h after virus adsorption that IE1 signals did not markedly differ between ΔUL50C and ΔUL50N (Figure 6D, left panels). In contrast, starting with the time point of 4 h, and even more drastically exerted at 8 h after virus adsorption, a pronounced reduction in the onset of IE1 expression was noted for ΔUL50N (Figure 6D, right panels). This is specifically illustrated by the values of IE1-positive cells of 11% and 14% compared to 8% and 6% at 4 h, as well as 61% and 50% compared to 2% and 2% at 8 h, respectively. Notably, the state of +dox or -dox induction did not substantially influence this difference between the ΔUL50N and ΔUL50C viruses. 

In an additional confocal imaging analysis, in which the IE1 expression pattern was monitored in infected cells beyond time points of 24 h, IE1-positive cells were comparably immunostained in ΔUL50C- and ΔUL50N-infected cells (Figure S3). In both cases, IE1 expression levels remained constant over the time period of 1–8 d p.i. without dox induction (panel A: images 4, 12, 20 or images 8, 16, 24, respectively), whereby the overall quantity of infected cells was higher for ΔUL50C than for ΔUL50N (panels B–C). Upon dox induction, the strong increase in IE1 almost attained WT levels in the case of ΔUL50C until 8 d p.i. (panel B: 38.6 ± 2.1, 88.7 ± 7.5, 100.0 ± 0.0% of total cells at 1, 4, 8 d p.i., respectively). In contrast, ΔUL50N-infected cells showed a profoundly lower level of IE1 expression under dox induction within this time frame (panel B; 0.4 ± 0.7, 2.6 ± 2.5, 42.1 ± 7.0% of total cells at 1, 4, 8 d p.i., respectively). In comparison, the control IE1 expression curves of WT virus were found dox-independent as expected (panel C). This finding further illustrates the low number of infectious virions contained in ΔUL50N preparations compared to ΔUL50C (see Figure 5A). Thus, the data support that the particle preparations used as inocula of infection mainly contained genome-lacking NIEPs. In the case of ΔUL50N- infected cells, the number of IE1 expressions increased only under pUL50-complementing conditions over the course of time. Whereas, under non-complementing conditions, the number of IE1-expressing cells remained constant (Figure S3, panels A–C). In this low number of IE1-positive cells (mAb-IE1), additional data clearly demonstrated the progression of viral lytic replication into the early (E/pUL44) and late phases (L/pp150 and pp28; Figure S4). When monitoring the production of viral proteins pUL44 (panels A–B), pp150 and pp28 (panels C–D or E–F, respectively) at 4 d p.i., no qualitative difference (see merge images in the +dox settings shown in A, C, and E), but exclusively a quantitative difference was noted between ΔUL50N and ΔUL50C (see lower magnification in -dox settings shown in B, D and F). Thus, the combined findings clearly point to the fact that the ΔUL50N virus, produced under pUL50-negative conditions, is strongly limited in the quantity of infectious virions. In order to address the question, whether this deficiency was based on differences referring to the level of viral genomes, a genome-specific analysis of particles was performed.

### 3.7. Identification of Reduced Levels of Genomic DNA Packaging in ΔUL50N Particles

**Figure 7:**
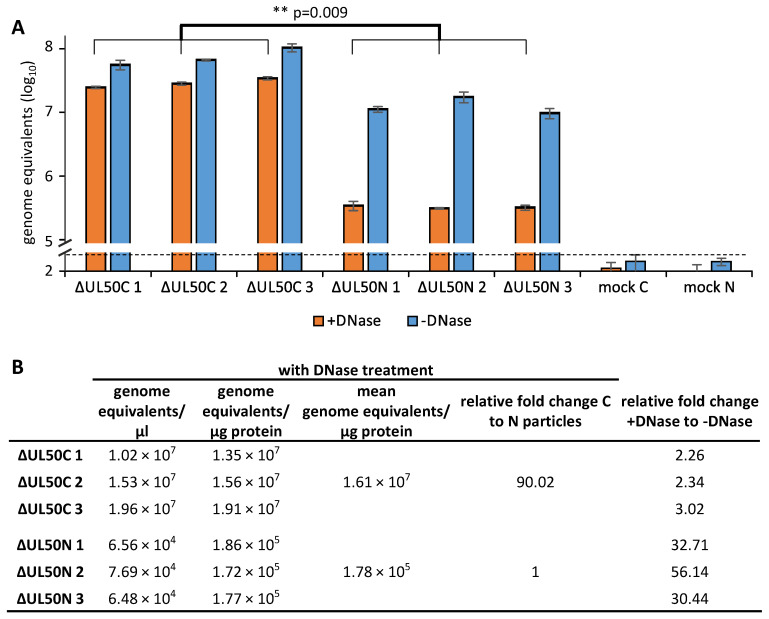
Purified ΔUL50N particles comprise a reduced quantity of viral genome packaging, and an association with DNase-sensitive unpackaged genomes. (**A**) Samples of sucrose cushion-purified ΔUL50C and ΔUL50N particles were adjusted to identical protein levels and either subjected to DNase digestion (+DNase) or remained untreated (−DNase) prior to the isolation of viral genomic DNA. In both cases, viral genomic loads were determined by virus-specific qPCR. Mean values of triplicates ± SD are given. Statistical significance between the mean values of ΔUL50C 1–3 and ΔUL50N 1–3 was calculated by the Student’s *t*-test (**, *p* = 0.009). (**B**) Calculation of genome equivalents per μg protein in the respective aliquots. Relative fold change between the mean values of ΔUL50C and ΔUL50N particles and between the settings with (+) and without (−) DNase treatment are given. Note the drastic change in the detection of genome equivalents after DNase treatment (+) determined for the ΔUL50N virus samples.

To address the question of whether the levels of genomic DNA packaging might show differences between ΔUL50C and ΔUL50N particles, these were subjected to DNase digestion prior to the qPCR analysis, in order to eliminate unpackaged viral genomes from the measurements (Figure 7). To this end, the ΔUL50 samples were adjusted to identical protein levels. As a very central result, this qPCR analysis showed that ΔUL50N particles comprised substantially reduced levels of encapsidated genomic DNA compared to ΔUL50C (Figure 7A, orange bars). In both cases, with and without DNase treatment, the amounts of viral genomes varied to some extent when comparing the ΔUL50C and ΔUL50N particles (+DNase *p* = 0.009; −DNase *p* = 0.047). Most importantly, the differences between the settings with and without DNase for the particles ΔUL50C and ΔUL50N were finally expressed as quantitative mean values of genomic equivalents (Figure 7B). These data showed that ΔUL50C particles contained 90.02-fold increased viral genomes per μg protein than ΔUL50N particles. Moreover, the relative fold change between the two settings with and without DNase showed clear differences, thus strongly suggesting an association of unpackaged viral genomes with ΔUL50 particles (Figure 7B; note the relative fold change of 2.26–3.02 +DNase/−DNase for ΔUL50C, as compared to 30.44–56.14 for ΔUL50N). Thus, these data indicate that ΔUL50N particles comprised reduced levels of packaged genomic DNA, but high levels of unpackaged genomic DNA, in comparison to ΔUL50C. These data support the concept that deficiencies in the degree of infectivity of ΔUL50N particles are most probably based on defects in the correct packaging of viral genomes.

## 4. Conclusions

The nuclear egress represents a rate-limiting step during the lytic replication and production of infectious progeny of all herpesviruses. HCMV pUL50 and pUL53, like their respective herpesviral homologs, are the core NEC components and major egress regulators. Although the main functions, structural features, and binding properties of these core NEC proteins have been well recognized, several accessory activities have been reported, in particular for the pUL50 groove protein homologs [50,51,52,53,54,55]. In the present study, we focused on directly NEC-dependent and partly NEC-independent functional aspects of HCMV pUL50 through the characterization of the HCMV ΔUL50 deletion mutant. Surprisingly, as reported recently, this mutant is able to transiently maintain a very low level of lytic reproduction, before the pUL50 defect ultimately terminates the course of infection [33]. Virus propagation was facilitated by the use of pUL50- complementing cells, and we observed a nuclear accumulation of immature A-type capsids occurring under non-complementing -dox conditions, while B- and C-type capsids were found underrepresented [13]. The high abundance of A-type capsids directly relates to the finding of abortive genome packaging described in the present study. Here, we performed a more detailed phenotypical characterization of virions produced either in the presence of pUL50 (ΔUL50C preparations), or in its absence (ΔUL50N), in order to detect putative pUL50-specific effects that may reach beyond the regulation of nuclear egress. Indeed, we were able to identify distinct features of the ΔUL50N virus preparations pointing to the fact that pUL50 fulfills a regulatory role that goes beyond the classical nuclear egress functions: (i) ΔUL50N particles comprised a drastically more pronounced replicative defect than ΔUL50C particles; (ii) gradient purifications even demonstrated a lack of detectable virion fraction for ΔUL50N compared to ΔUL50C; (iii) mass spectro- metry-based and immuno-EM analyses showed a large concordance of proteomic contents in the detectable particle fractions (NIEPs) between ΔUL50C and ΔUL50CN, and did not reveal major disorders in EM nuclear protein distributions, respectively; (iv) confocal imaging indicated a quantitatively limited onset of viral lytic protein expression in the initially very low number of infected cells; and (v) the quantitative assessment of encapsidated genomes provided evidence for a substantial reduction in ΔUL50N compared to ΔUL50C. The latter finding, in particular, strongly supported our earlier concept that the ΔUL50N-specific deficiencies are primarily based on defects in the correct packaging of viral genomes. It should be noted that similar experiments were also performed with a protein quantitation-based adjustment of viral inocula (data not shown), instead of a genome quantitation-based adjustment. Importantly, also in those experiments, the determination of viral replication kinetics by qPCR confirmed the defect of ΔUL50N towards ΔUL50C. Nevertheless, one general limitation is seen in the challenge to define a uniform quantitative basis of inocula, on the one hand, and differences in the quantity of encapsidated genomes, on the other. For this reason, the central point of a future study should be the normalization of virus inocula quantities, i.e., ΔUL50N and ΔUL50C, determined by qPCR after DNase treatment for a further characterization of the functional role of pUL50. The DNase treatment appears to be a rather unusual practice, which has not been performed in our previous phenotypic characterization of mutant HCMVs so far [13,56,57,58,59]. However, the results of the present study suggest to take into account, specifically for ΔUL50 preparations, the different options of inocula normalization, such as total virion protein, virion DNA, encapsidated virion DNA, or plaque- forming units. Thus, the main conclusion derived from the experimental approaches of this study states that the efficiency of genome packaging is reduced in absence of pUL50. Combined, this points to a complex regulatory role of HCMV pUL50, which may extend beyond its so far considered NEC functions, in terms of viral maturation and the production of infectious progeny virus.

## Figures and Tables

**Table 1:** Classes of HCMV structural proteins contained in ΔUL50C and ΔUL50N NIEP fractions characterized by MS-based quantitative proteomics *.

Gene Name	Uniprot	Protein Name	Normalized iBAQ ΔUL50N		Normalized iBAQ ΔUL50C	
			Mean	SD	Mean	SD
**Capsid**						
MCP/UL86	P16729	Major capsid protein	1.04 × 10^7^	5.96 × 10^4^	1.06 × 10^7^	1.15 × 10^5^
SCP/UL48A	Q7M6N6	Smallest capsid protein	5.43 × 10^5^	2.06 × 10^5^	4.76 × 10^5^	1.67 × 10^5^
TRX1//UL46	P16783	Triplex capsid protein 1	2.16 × 10^6^	5.96 × 10^4^	1.96 × 10^6^	1.15 × 10^5^
TRX2/UL85	P16728	Triplex capsid protein 2	6.95 × 10^6^	1.10 × 10^5^	7.09 × 10^6^	8.11 × 10^5^
CVC1/UL93	P16799	Capsid vertex component 1	3.37 × 10^4^	9.64 × 10^3^	4.56 × 10^4^	1.15 × 10^4^
CVC2/UL77	P16729	Capsid vertex component 2	4.12 × 10^5^	3.66 × 10^4^	4.54 × 10^5^	8.14 × 10^4^
PORT/UL104	P16735	Portal protein	5.30 × 10^5^	1.30 × 10^5^	3.81 × 10^5^	9.03 × 10^4^
UL80	P16753	Capsid scaffolding protein	1.49 × 10^6^	1.92 × 10^5^	1.57 × 10^6^	2.03 × 10^5^
UL80.5	B8YEA6	Capsid scaffold protein	2.75 × 10^5^	1.07 × 10^5^	2.83 × 10^5^	6.45 × 10^4^
**Envelope**						
gB/UL55	P06473	Envelope glycoprotein B	1.30 × 10^7^	2.41 × 10^6^	1.06 × 10^7^	6.73 × 10^5^
gH/UL75	P12824	Envelope glycoprotein H	5.68 × 10^6^	8.15 × 10^5^	5.01 × 10^6^	3.23 × 10^5^
gL/UL115	P16832	Envelope glycoprotein L	1.51 × 10^6^	2.14 × 10^5^	1.31 × 10^6^	9.78 × 10^4^
gM/UL100	P16733	Envelope glycoprotein M	2.52 × 10^7^	7.47 × 10^6^	2.15 × 10^7^	1.29 × 10^6^
gN/UL73	P16795	Envelope glycoprotein N	3.39 × 10^6^	1.21 × 10^6^	1.96 × 10^6^	1.84 × 10^5^
gO/UL74	P16750	Glycoprotein O	2.45 × 10^5^	1.44 × 10^4^	1.47 × 10^5^	3.66 × 10^4^
UL132	P69338	Envelope glycoprotein UL132	6.71 × 10^6^	9.39 × 10^5^	5.89 × 10^6^	1.37 × 10^5^
UL41A	O39920	Protein UL41A	5.35 × 10^6^	8.62 × 10^5^	5.09 × 10^6^	2.86 × 10^5^
TRL10/RL10	P16808	Protein IRL10	1.37 × 10^6^	2.33 × 10^5^	1.19 × 10^6^	7.28 × 10^4^
UL119/UL118	P16739	Viral Fc-gamma receptor-like protein UL119	3.31 × 10^5^	9.34 × 10^4^	4.05 × 10^5^	3.00 × 10^4^
UL116	P16833	Uncharacterized protein UL116	7.65 × 10^5^	1.12 × 10^5^	6.60 × 10^5^	8.10 × 10^4^
UL128	P16837	Uncharacterized protein UL128	4.53 × 10^5^	1.15 × 10^5^	5.46 × 10^5^	1.59 × 10^5^
**Tegument**						
pp65/UL83	P06725	65 kDa phosphoprotein	1.01 × 10^8^	2.23 × 10^7^	9.41 × 10^7^	9.45 × 10^6^
pp85/UL25	P16761	Phosphoprotein 85	7.31 × 10^6^	1.57 × 10^6^	6.14 × 10^6^	8.48 × 10^5^
pp71/UL82	P06726	Protein pp71	1.32 × 10^7^	2.21 × 10^6^	1.10 × 10^7^	6.57 × 10^5^
pp150/UL32	P08318	Large structural phosphoprotein	6.34 × 10^6^	1.33 × 10^6^	5.01 × 10^6^	2.08 × 10^5^
UL103	P16734	Cytoplasmic envelopment protein 1	5.75 × 10^6^	7.45 × 10^5^	5.53 × 10^6^	1.72 × 10^5^
UL94	P16800	Cytoplasmic envelopment protein 2	7.72 × 10^6^	1.28 × 10^6^	6.99 × 10^6^	4.04 × 10^5^
pp28/UL99	P13200	Cytoplasmic envelopment protein 3	7.58 × 10^6^	1.84 × 10^6^	6.79 × 10^6^	2.25 × 10^5^
UL47	P16784	Inner tegument protein	4.83 × 10^6^	6.54 × 10^5^	3.99 × 10^6^	1.93 × 10^5^
UL48	P16785	Large tegument protein deneddylase	4.16 × 10^6^	8.22 × 10^5^	3.04 × 10^6^	7.10 × 10^4^
UL71	P16823	Tegument protein UL51 homolog	2.32 × 10^6^	1.27 × 10^5^	2.24 × 10^6^	1.70 × 10^5^
UL26	P16762	Tegument protein UL26	3.50 × 10^6^	7.42 × 10^5^	2.89 × 10^6^	1.98 × 10^5^
UL43	P16781	Tegument protein UL43	8.14 × 10^5^	7.89 × 10^4^	8.34 × 10^5^	1.18 × 10^5^
US24	P09700	Tegument protein US24	3.94 × 10^4^	4.62 × 10^4^	2.91 × 10^4^	2.06 × 10^4^
RIR1/UL45	P16782	Ribonucleoside-diphosphate reductase large subunit-like protein	5.11 × 10^6^	1.08 × 10^6^	4.71 × 10^6^	1.91 × 10^5^
US23	B8YEI2	Protein US23	3.04 × 10^4^	1.73 × 10^4^	2.56 × 10^4^	1.26 × 10^4^
UL88	P16731	Protein UL88	2.93 × 10^6^	1.93 × 10^5^	2.20 × 10^6^	1.93 × 10^5^
UL24	P16760	Protein UL24	2.53 × 10^6^	4.18 × 10^5^	2.16 × 10^6^	2.25 × 10^5^
UL35	P16766	Protein UL35	1.96 × 10^6^	3.52 × 10^5^	1.60 × 10^6^	2.17 × 10^5^
UL96	P16787	Protein UL96	1.04 × 10^5^	1.29 × 10^5^	1.85 × 10^5^	4.68 × 10^4^
TRS1	P09695	Protein HHLF1	9.47 × 10^5^	1.25 × 10^4^	8.73 × 10^5^	9.11 × 10^4^
IRS1	P09715	Protein IRS1	4.88 × 10^6^	3.27 × 10^5^	4.61 × 10^6^	3.53 × 10^5^
UL97	P16788	Serine/threonine protein kinase UL97	1.32 × 10^6^	2.32 × 10^5^	1.20 × 10^6^	1.06 × 10^5^
UL69	P16749	mRNA export factor ICP27 homolog	1.02 × 10^6^	2.49 × 10^4^	8.64 × 10^5^	9.03 × 10^4^
US22	P09722	Early nuclear protein HWLF1	9.38 × 10^6^	5.17 × 10^5^	7.52 × 10^6^	9.03 × 10^5^

* Proteins identified in biological triplicates of ΔUL50C and ΔUL50N NIEP fractions are grouped according to their localization within the virion. The iBAQ values of all detected proteins were normalized by adjusting the values to the mean iBAQ values of MCP and TRX1.

## Data Availability

The responsible authors declare that this article fully complies with the Data Availability Statements in section “MDPI Research Data Policies” at https://www.mdpi.com/ethics.

## Supplementary Materials

The following are available online at https://www.mdpi.com/article/10.3390/cells10113119/s1. Figure S1: Analysis of viral protein and genome contents of purified ΔUL50 particles. Figure S2: Histograms illustrating the distribution and variance of the individual values of the measurements. Figure S3: Quantitative evaluation of IE1 expression in ΔUL50C- and ΔUL50N-infected HFF-UL50. Figure S4: Confocal IF detection of viral IE, E and L proteins in ΔUL50C- and ΔUL50N-infected HFF-UL50. Table S1: Differential proteomic analysis of NIEPS fractions enriched from pUL50-complementing cells (ΔUL50C) or non-complementing cells (ΔUL50N). Table S2: Quantitative proteomic analysis of virions purified from pUL50-complementing cells (ΔUL50C).
