# Supplementary material for: The Complex Regulatory Role of Cytomegalovirus Nuclear Egress Protein pUL50 in the Production of Infectious Virus
Source: Cells. 2021 Nov 11;10(11):3119. doi: 10.3390/cells10113119 (PMC8625744; doi:10.3390/cells10113119)
Supplement: Supplementary file 1 [file cells-10-03119-s001.zip › cells-1432339-Supplementary Materials.pdf]

cells

MDPI

Supplementary Materials

The Complex Regulatory Role of Cytomegalovirus Nuclear Egress Protein pUL50 in the Production of Infectious Virus

Sigrun Häge [1,*], Nicole Büscher [2], Victoria Pakulska [3], Friedrich Hahn [1], Annie Adrait [3], Steffi Krauter [2], Eva Maria Borst [4], Ursula Schlötzer-Schrehardt [5], Yohann Couté [3], Bodo Plachter [2] and Manfred Marschall [1,*]

1 Institute for Clinical and Molecular Virology, Friedrich-Alexander University of Erlangen-Nürnberg (FAU), 91054 Erlangen, Germany; sigrun.haege@fau.de (S.H.); friedrich.hahn@uk-erlangen.de (F.H.); manfred.marschall@fau.de (M.M.)
2 Institute for Virology and Forschungszentrum für Immuntherapie, University Medical Center of the Johannes Gutenberg-University Mainz, 55131 Mainz, Germany; bueschni@uni-mainz.de (N.B.); krauter@uni-mainz.de (S.K.); plachter@uni-mainz.de (B.P.)
3 University Grenoble Alpes, INSERM, CEA, UMR BioSanté U1292, CNRS, CEA, FR2048, 38000 Grenoble, France; victoria.pakulska@cea.fr (V.P.); annie.adrait@cea.fr (A.A.); yohann.coute@cea.fr (Y.C.)
4 Institute of Virology, Hannover Medical School (MHH), Hannover, Germany; borst.eva@mh-hannover.de (E.M.B.)
5 Department of Ophthalmology, University Medical Center Erlangen, FAU, 91054 Erlangen, Germany; Ursula.Schloetzer-Schrehardt@uk-erlangen.de (U.S.S.)
* Correspondence: sigrun.haege@fau.de (S.H.), manfred.marschall@fau.de (M.M.); phone: +49-9131-8526089

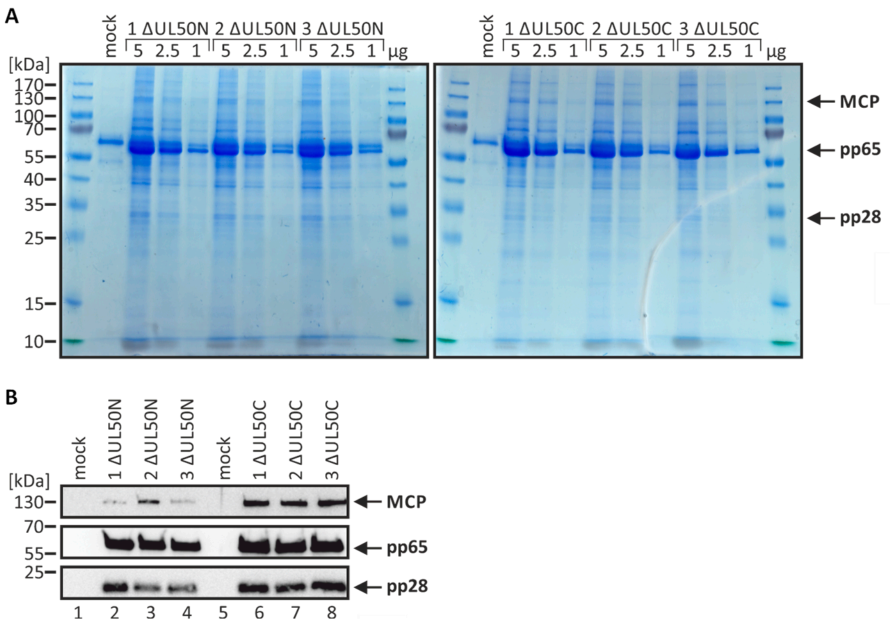

C

	genomes/µl	protein in µg/µl	mean genomes	mean proteins	factor genomes	factor proteins
**ΔUL50N 1**	2.98×10^{5}	0.35	5.15×10^{5} $\pm 5.26 \times 10^{5}$	0.39 ± 0.05	1	1
**ΔUL50N 2**	1.11×10^{6}	0.45				
**ΔUL50N 3**	1.32×10^{5}	0.37				
**ΔUL50C 1**	3.06×10^{6}	0.75	4.93×10^{6} $\pm 1.85 \times 10^{6}$	0.92 ± 0.14	9.6	2.4
**ΔUL50C 2**	4.96×10^{6}	0.98				
**ΔUL50C 3**	6.76×10^{6}	1.03				

Figure S1: Analysis of viral protein and genome contents of purified ΔUL50 particles. (A) Instant blue staining of ΔUL50C and ΔUL50N particles using identical concentrations of protein. **(B)** Wb-based detection of viral proteins contained in purified particles using the indicated antibodies. **(C)** Viral genomic equivalents as genomes/µl, measured by IE1-specific qPCR, and protein concentrations as µg/µl measured by BCA assay. Mean values ± SD and factor-fold changes between ΔUL50C and ΔUL50N particles are given. Samples 1, 2 and 3 represent triplicates of the purification.

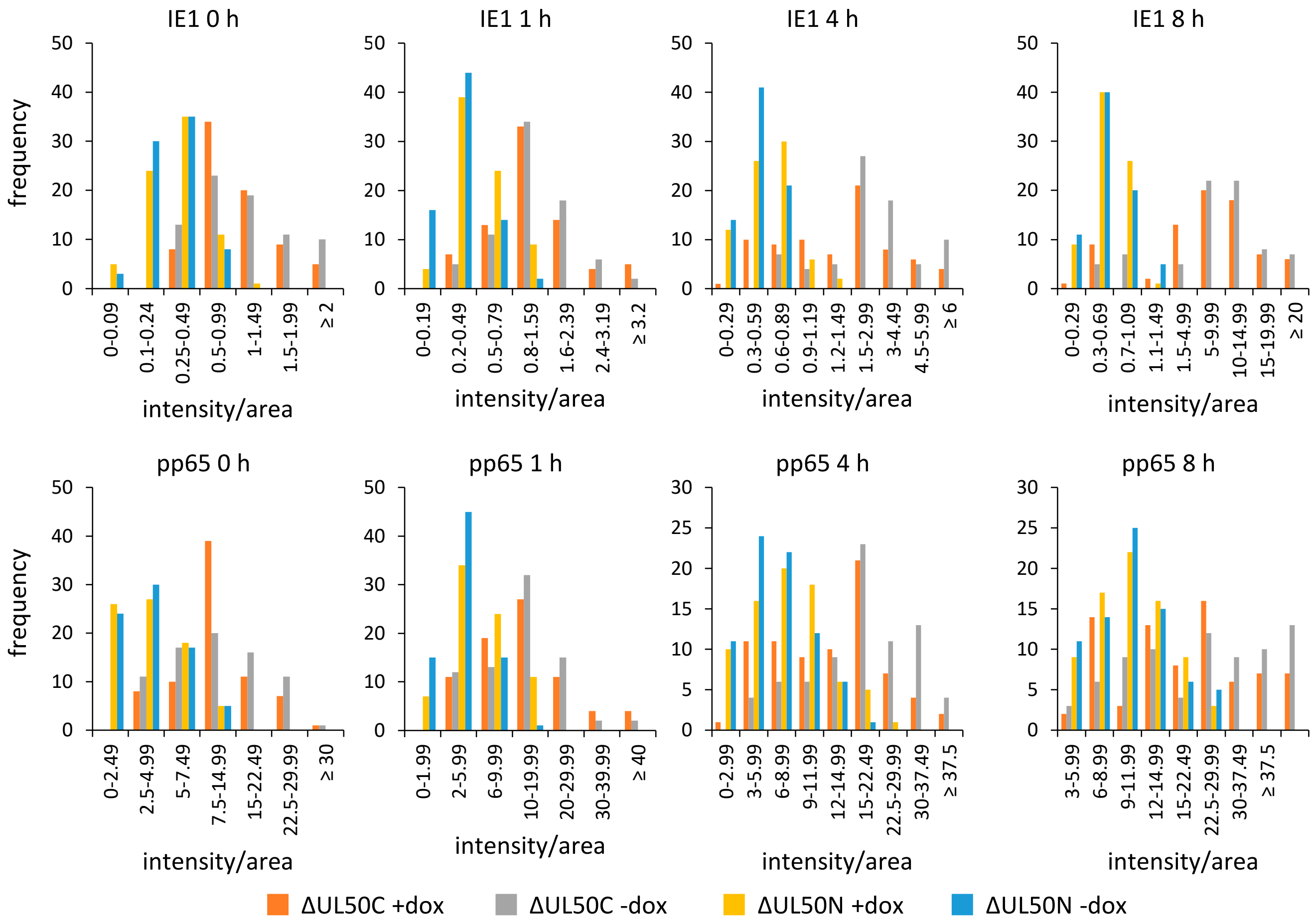

Figure S2: Histograms illustrating the distribution and variance of the individual values of the measurements. All values of the quantitative evaluation of the confocal IF imaging shown in Figure 6B-C were used for a dissection of groups of values into separate classes of signal intensities of IE1 and pp65 as indicated.

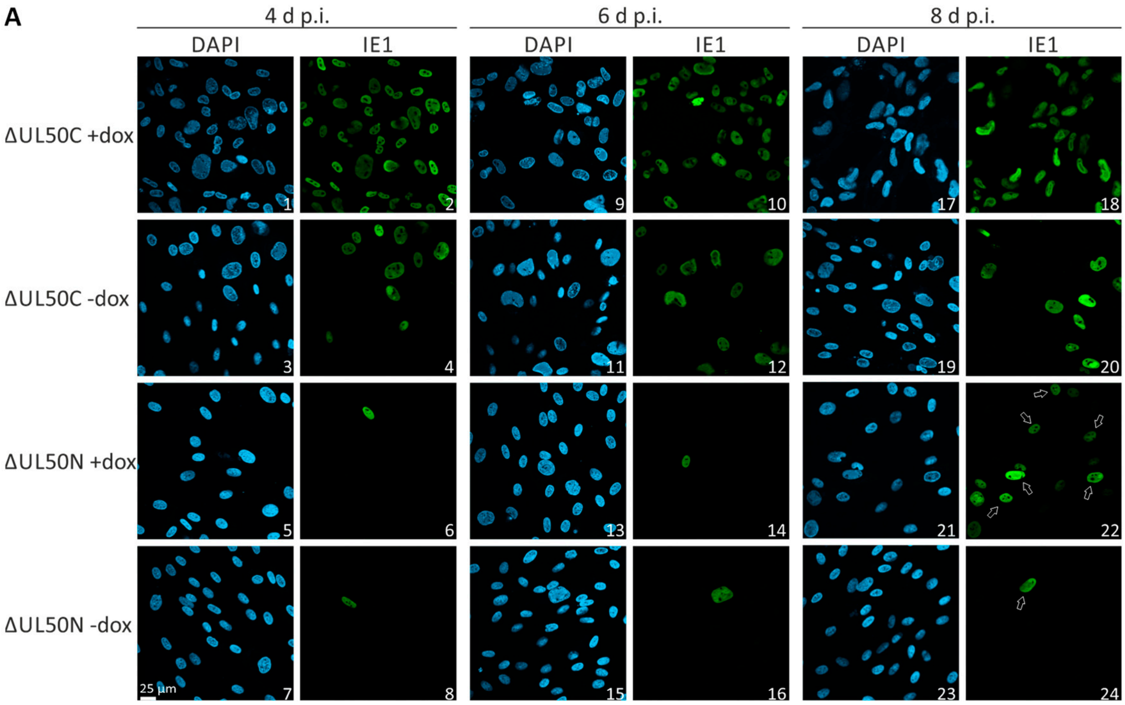

B

	1 d p.i.	4 d p.i.	8 d p.i.
WT +dox	61.0 ± 3.9	100.0 ± 0.0	100.0 ± 0.0
WT -dox	66.7 ± 10.8	100.0 ± 0.0	100.0 ± 0.0
ΔUL50C +dox	38.6 ± 2.1	88.7 ± 7.5	100.0 ± 0.0
ΔUL50C -dox	29.8 ± 4.8	29.7 ± 7.9	28.1 ± 2.8
ΔUL50N +dox	0.4 ± 0.7	2.6 ± 2.5	42.1 ± 7.0
ΔUL50N -dox	0.5 ± 0.9	0.3 ± 0.6	0.5 ± 0.9

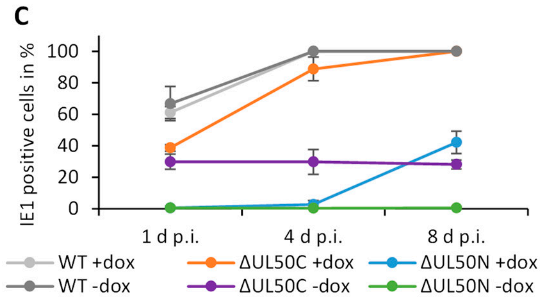

Figure S3: Quantitative evaluation of IE1 expression in ΔUL50C- and ΔUL50N-infected HFF-UL50. (A) HFF-UL50, either induced or uninduced, were infected with WT, ΔUL50C or ΔUL50N, adjusted to identical viral genome copy numbers. Cells were fixed at the indicated time points and used for immunostaining with an IE1-specific antibody and subsequent analysis by confocal imaging. The nuclei were counterstained by DAPI, scale bar is given in 7. **(B)** At least 50 cells were counted in triplicates. Mean values ± SD of IE1 positive cells are given, as set in percentage to the total number of cells counted. **(C)** Diagram representation of the mean values given in B.

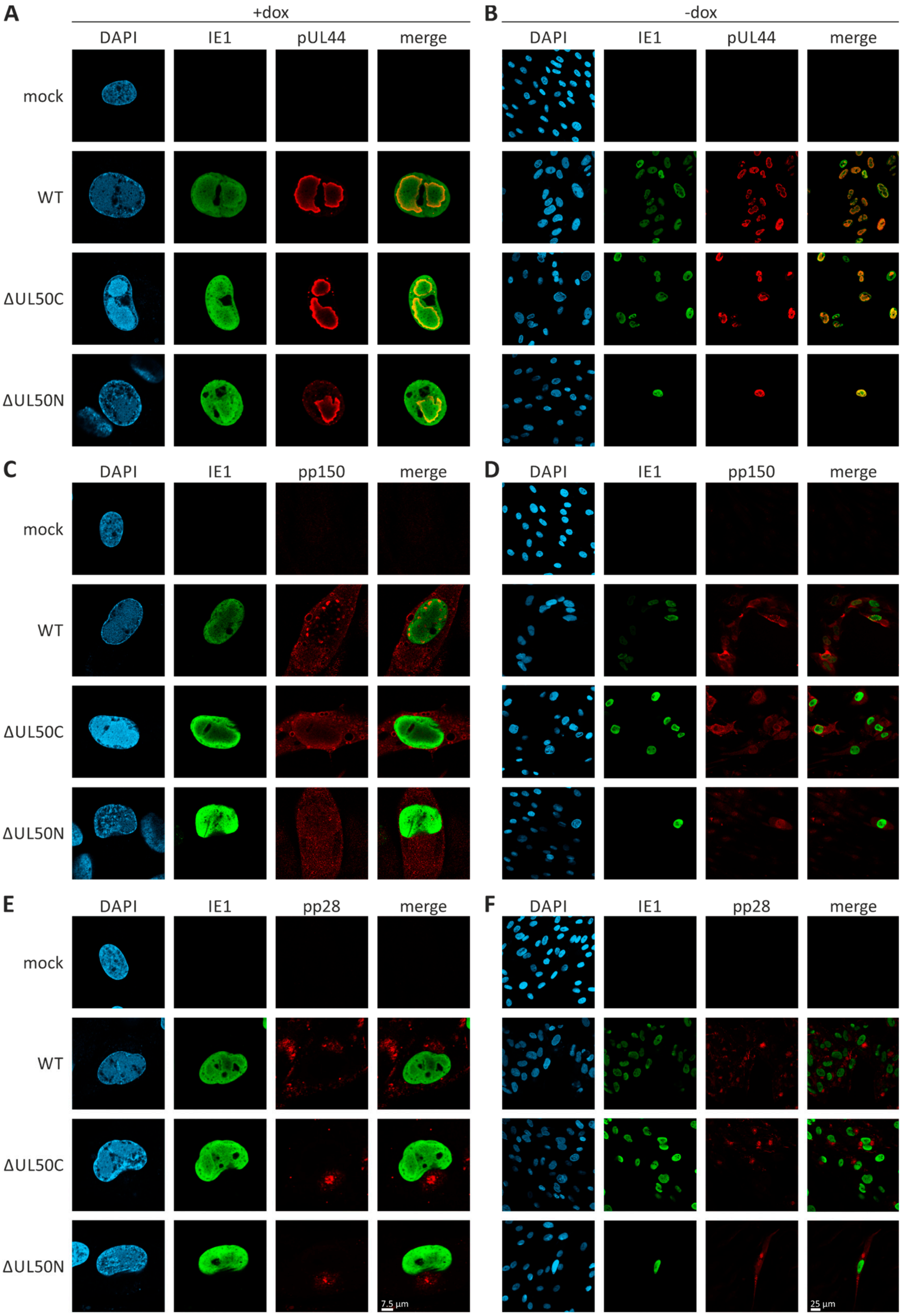

Figure S4: Confocal IF detection of viral IE, E and L proteins in ΔUL50C- and ΔUL50N-infected HFF-UL50. HFF-UL50, either dox-induced **(A, C, E)** or uninduced **(B, D, F)**, were infected with WT, ΔUL50C or ΔUL50N, as adjusted to identical viral genome copy numbers. Cells were fixed at 4 d p.i. and used for immunostaining with virus-specific antibodies and subsequent analysis by confocal imaging. Expression of the immediate early protein IE1 **(A-F)**, early protein pUL44 **(A, B)**, and the late proteins pp150 **(C, D)** as well as pp28 **(E, F)**. The nuclei were counterstained by DAPI; merge represents an overlay of the green and red signals; scale bars are given in the lower panels.

Table S1: Differential proteomic analysis of NIEPS fractions enriched from pUL50-complementing cells (ΔUL50C) or non-complementing cells (ΔUL50N).

Table S2: Quantitative proteomic analysis of virions purified from pUL50-complementing cells (ΔUL50C).
